# COVID-19 Vaccine Hesitancy on Social Media: Building a Public Twitter Data Set of Antivaccine Content, Vaccine Misinformation, and Conspiracies

**DOI:** 10.2196/30642

**Published:** 2021-11-17

**Authors:** Goran Muric, Yusong Wu, Emilio Ferrara

**Affiliations:** 1 Information Sciences Institute University of Southern California Marina del Rey, CA United States; 2 Department of Computer Science University of Southern California Los Angeles, CA United States; 3 Annenberg School for Communication and Journalism University of Southern California Los Angeles, CA United States

**Keywords:** vaccine hesitancy, COVID-19 vaccines, dataset, COVID-19, SARS-CoV-2, social media, network analysis, hesitancy, vaccine, Twitter, misinformation, conspiracy, trust, public health, utilization

## Abstract

**Background:**

False claims about COVID-19 vaccines can undermine public trust in ongoing vaccination campaigns, posing a threat to global public health. Misinformation originating from various sources has been spreading on the web since the beginning of the COVID-19 pandemic. Antivaccine activists have also begun to use platforms such as Twitter to promote their views. To properly understand the phenomenon of vaccine hesitancy through the lens of social media, it is of great importance to gather the relevant data.

**Objective:**

In this paper, we describe a data set of Twitter posts and Twitter accounts that publicly exhibit a strong antivaccine stance. The data set is made available to the research community via our AvaxTweets data set GitHub repository. We characterize the collected accounts in terms of prominent hashtags, shared news sources, and most likely political leaning.

**Methods:**

We started the ongoing data collection on October 18, 2020, leveraging the Twitter streaming application programming interface (API) to follow a set of specific antivaccine-related keywords. Then, we collected the historical tweets of the set of accounts that engaged in spreading antivaccination narratives between October 2020 and December 2020, leveraging the Academic Track Twitter API. The political leaning of the accounts was estimated by measuring the political bias of the media outlets they shared.

**Results:**

We gathered two curated Twitter data collections and made them publicly available: (1) a streaming keyword–centered data collection with more than 1.8 million tweets, and (2) a historical account–level data collection with more than 135 million tweets. The accounts engaged in the antivaccination narratives lean to the right (conservative) direction of the political spectrum. The vaccine hesitancy is fueled by misinformation originating from websites with already questionable credibility.

**Conclusions:**

The vaccine-related misinformation on social media may exacerbate the levels of vaccine hesitancy, hampering progress toward vaccine-induced herd immunity, and could potentially increase the number of infections related to new COVID-19 variants. For these reasons, understanding vaccine hesitancy through the lens of social media is of paramount importance. Because data access is the first obstacle to attain this goal, we published a data set that can be used in studying antivaccine misinformation on social media and enable a better understanding of vaccine hesitancy.

## Introduction

The opposition to vaccination dates back to the 1800s, immediately after the English physician Edward Jenner created the first vaccine in human history. The opponents to the vaccine were vocal and could be found in all segments of society: religious communities protested the unnaturalness of using animal infection in humans, parents were concerned about the invasiveness of the procedure, and vaccinated people were often illustrated with a cow’s head growing from their neck [1]. Although vaccination is an effective way to prevent diseases such as diphtheria, tetanus, pertussis, influenza, and measles, almost 1 in 5 children still do not receive routine lifesaving immunizations, and an estimated 1.5 million children still die each year of diseases that could be prevented by vaccines that already exist [2]. These fatalities are not only caused by objective reasons, such as lack of access to vaccines due to poverty, but also by the unwillingness and fear regarding vaccines from the parents of these children. The term “vaccine hesitancy” refers to delay in acceptance or refusal of vaccines despite availability of vaccine services [3]. Vaccine hesitancy has emerged as a factor in vaccine delay and refusal for adults. A common example is the annual seasonal influenza vaccine. It has been observed that greater hesitancy, both general and specific to the influenza vaccine, is associated with lower vaccine uptake [4,5]. A variety of factors contribute to vaccine hesitancy, including safety concerns, religious reasons, personal beliefs, philosophical reasons, and desire for additional education [6]. During the COVID-19 pandemic, although the inoculation of large populations is increasingly important, antivaccine narratives are spreading rapidly, endangering public health, human lives, and the social order.

With the rise of social media, the dissemination of information (and hence, potentially, misinformation) has become easier than ever before. Unsurprisingly, antivaccine activists have also begun to use platforms such as Twitter to share their views. As a result, their activism has expanded its jurisdictions to include web-based propaganda. Compared with traditional communication channels, social media offers an unprecedented opportunity to spread antivaccination messages and allow communities to form around antivaccine sentiment [7]. Social media can amplify the effects of antivaccination misinformation; multiple studies have shown links between susceptibility to misinformation and both vaccine hesitancy and a reduced likelihood to comply with health guidance measures [7-10]. Based on these findings, vaccine-related misinformation on social media may exacerbate the levels of vaccine hesitancy, creating pockets with low vaccination rates in the United States and globally; this can hamper progress toward vaccine-induced herd immunity and can potentially increase the number of infections related to new COVID-19 variants, possibly leading to vaccine-resistant mutations. For these reasons, understanding vaccine hesitancy through the lens of social media is of paramount importance. Because data access is the first obstacle to attain this goal, to enable the research community, we built and made public a social media data set of antivaccine content, vaccine misinformation, and related conspiracies. Although researchers have been collecting data related to COVID-19 vaccines [11], per our knowledge, there are no public data sets focused specifically on the historical activities of antivaccination accounts on Twitter.

Here, we present a data set that focuses on antivaccine narratives on Twitter. The data set consists of two complementary collections: (1) the *streaming collection* contains tweets collected using the Twitter Streaming application programming interface (API) from a set of antivaccine keywords, and (2) the *account collection* contains historical tweets from approximately 70,000 accounts that engaged in spreading antivaccination narratives. Additionally, we present initial statistical analyses of the data, including the frequencies of hashtags, analysis of the news sources, the most likely political leaning of the accounts, and geographic distribution.

The published data set includes tweet IDs of publicly available posts, in compliance with the Twitter Terms of Service [12]. This collection builds on the previously published data sets by DeVerna et al [11], which is focused on general vaccine narratives, and it complements the previous work by Chen et al [13] and Lamsal [14], who published some of the largest Twitter data sets related to COVID-19 discourse to date. The complete data set in the form of a list of tweet IDs is openly available on GitHub [15].

## Methods

### Tracked Keywords for the Streaming Collection

To create a set of keywords that indicate opposition to vaccines, we used a snowballing sampling technique similar to that of DeVerna et al [11]. We started from a small set of manually curated keywords used exclusively in the context of strong vaccine hesitancy that appear on Twitter, such as *#vaccineskill* or *#vaccinedamage*. Using the Twitter Streaming API and the set of seed keywords, we collected the data for one day (October 18, 2020), after which we extracted other keywords that co-occurred with the seed keywords. We added the newly collected keywords to the list of seed keywords, checking them manually for relevance. We then repeated this step several times until we exhausted all the significant co-occurrences and narrowed our selection to approximately 60 keywords. The Twitter API can be queried with a substring of a longer keyword, and it will return the tweets that contain the substring. For example, the keyword *novaccine* will return the tweets that contain *novaccineforme*. We attempted to retain only the most informative and relevant stem words to capture most vaccine-related tweets and to avoid collecting less relevant tweets. The list of all keywords used to collect the streaming collection is listed in Table 1.

**Table 1:** Set of keywords used to collect the tweets in the streaming collection.

Keyword	Date on which tracking began
*abolishbigpharma*	12/30/2020
*antivaccine*	12/30/2020
*ArrestBillGates*	10/19/2020
*betweenmeandmydoctor*	12/30/2020
*bigpharmafia*	10/19/2020
*bigpharmakills*	12/30/2020
*BillGatesBioTerrorist*	10/19/2020
*billgatesevil*	12/30/2020
*BillGatesIsEvil*	10/19/2020
*billgatesisnotadoctor*	12/23/2020
*billgatesvaccine*	12/14/2020
*cdcfraud*	10/19/2020
*cdctruth*	10/19/2020
*cdcwhistleblower*	10/19/2020
*covidvaccineispoison*	12/23/2020
*depopulation*	10/19/2020
*DoctorsSpeakUp*	10/19/2020
*educateb4uvax*	10/19/2020
*exposebillgates*	12/30/2020
*forcedvaccines*	12/30/2020
*Fuckvaccines*	10/19/2020
*idonotconsent*	12/30/2020
*informedconsent*	12/14/2020
*learntherisk*	10/19/2020
*medicalfreedom*	12/30/2020
*medicalfreedomofchoice*	12/30/2020
*momsofunvaccinatedchildren*	12/30/2020
*mybodymychoice*	12/30/2020
*noforcedflushots*	12/30/2020
*NoForcedVaccines*	10/19/2020
*notomandatoryvaccines*	12/30/2020
*NoVaccine*	10/19/2020
*NoVaccineForMe*	10/19/2020
*novaccinemandates*	12/30/2020
*parentalrights*	12/30/2020
*parentsoverpharma*	12/30/2020
*saynotovaccines*	12/30/2020
*stopmandatoryvaccination*	10/19/2020
*syringeslaughter*	12/30/2020
*unvaccinated*	12/30/2020
*v4vglobaldemo*	12/30/2020
*vaccinationchoice*	12/30/2020
*VaccineAgenda*	10/19/2020
*vaccinedamage*	10/19/2020
*vaccinefailure*	10/19/2020
*vaccinefraud*	10/19/2020
*vaccineharm*	10/19/2020
*vaccineinjuries*	12/30/2020
*vaccineinjury*	10/19/2020
*VaccinesAreNotTheAnswer*	10/19/2020
*vaccinesarepoison*	10/19/2020
*vaccinescause*	10/19/2020
*vaccineskill*	10/19/2020
*vaxxed*	11/02/2020
*yeht*	11/02/2020

### Collecting Tweets for Account Collection

First, we identified a randomly sampled set of approximately 70,000 accounts that appeared in the streaming collection and that engaged in antivaccine rhetoric between October and December 2020, either by tweeting some of the tracked keywords or by retweeting tweets that contained some of the tracked keywords. Then, for those accounts, we collected their historical tweets using the Twitter API. By leveraging Twitter's Academic Research product track, we were able to access the full archival search and overcome the limit of 3200 historical tweets of the standard API. In this way, we collected almost all the historical tweets of the most queried accounts.

Our collection relies upon publicly available data in accordance with the Content Redistribution clause under Twitter’s Developer Agreement and Policy [12]. We released the data set with the stipulation that those who use it must comply with Twitter’s Terms and Conditions. The complete data set is publicly available on a GitHub repository and is accessible on the web [15].

### Calculating the Political Leanings of the Accounts

We calculate the political leaning of each account by measuring the political bias of the media outlets it shared. We use a methodology proposed in prior work [16-18], and we identified a set of 90 prominent media outlets and accounts that appeared on Twitter. Each of these outlets and their associated Twitter accounts were placed on a political spectrum (left, lean left, center, lean right, right) per ratings provided by the nonpartisan service AllSides [19]. For each account in the data set, we maintained a record of all retweets and the original tweets that contained a domain name affiliated with the selected media outlets. The political bias of each account was calculated as the average political bias of all media outlets it shared content from.

### Identifying Low- and High-Credibility Media Sources

We leveraged *urllib*, the Python URL handling module, to parse the URLs found in the data set. Each URL was broken into several components, including the addressing scheme, network location, and path. A third-party data set that contains the domains associated with websites that share misinformation was used as a ground truth to tag the domain names [20]. For URLs that were not in the data set, we queried the Media Bias/Fact Check website [21] for further identification. Because URL shortening services such as Bitly [22] are widely used on Twitter, shortened URLs appeared frequently. We used *urlExpander* [23] to expand the shortened URLs and retrieve the full URLs where possible. Domain names of popular news aggregators and social networks such as Twitter, Facebook, Instagram, Periscope, and YouTube were ignored in the analysis.

### Generating Geolocation Distribution Maps

To infer a tweet’s geolocation, we used the information of the self-reported location of the account and matched it to a corresponding state in the United States. To calculate the average activity level per population, the absolute number of Tweets was normalized by the 2010 Census-reported population of that state as follows: I = *N_i_*/*P_i_* × 1,000,000, where *N_i_* is the number of tweets originating in state *i* and *P_i_* is that state’s population in 2010. This normalization provided information on the average number of collected tweets per million inhabitants. Note that we did not generate the geolocation map for the account collection, as it contains a relatively small number of accounts with self-reported locations.

### Topic Network Analysis

A topic network was constructed to analyze the co-occurrence of hashtags in the streaming data set. Each node in the graph represented a hashtag, and an edge was added if two hashtags occurred in the same tweet. The node size was proportional to its degree of centrality, and the edge weight was the number of times two hashtags appeared together. For better visualization, nodes with fewer than 25 neighbors were ignored. To investigate the community structure of the network, we used the Louvain algorithm [24] on the topic network, which provided further insights about the links between antivaccine topics.

## Results

The primary contribution of this study is the data set that we made publicly available. As of this writing (May 2021), we had collected over 137 million tweets organized in two collections. The streaming collection was gathered using the set of antivaccine keywords in Table 1. The account collection, on the other hand, contains the historical activities of accounts prone to spreading antivaccination narratives; thus, it is a significantly larger data set compared to the streaming collection. The basic statistics on the two data sets are shown in Table 2. The data set is available on GitHub [15] and was released in compliance with the Twitter Terms and Conditions. We are unable to provide the full text of the tweets; therefore, we are releasing the Tweet IDs, which are unique identifiers tied to specific tweets. Researchers can retrieve the full text and the related metadata by querying the Twitter API. Because the streaming data collection is still ongoing, the statistics shown below can vary in future versions of the data set. In the following sections, we will describe the streaming collection and account collection separately.

**Table 2:** Basic statistics on tweets collected in the streaming collection and account collection.

	Streaming collection	Account collection
Tweets, n	1,832,333	135,949,773
Accounts, n	719,652	78,954
Average number of tweets per account	2.5	1721.8
Verified accounts, n	9032	239
Accounts with location, n	5661	363
Date of oldest tweet	10/19/2020	3/6/2007
Date of most recent tweet	4/21/2021	2/2/2021

### Streaming Collection

**Figure 1:**
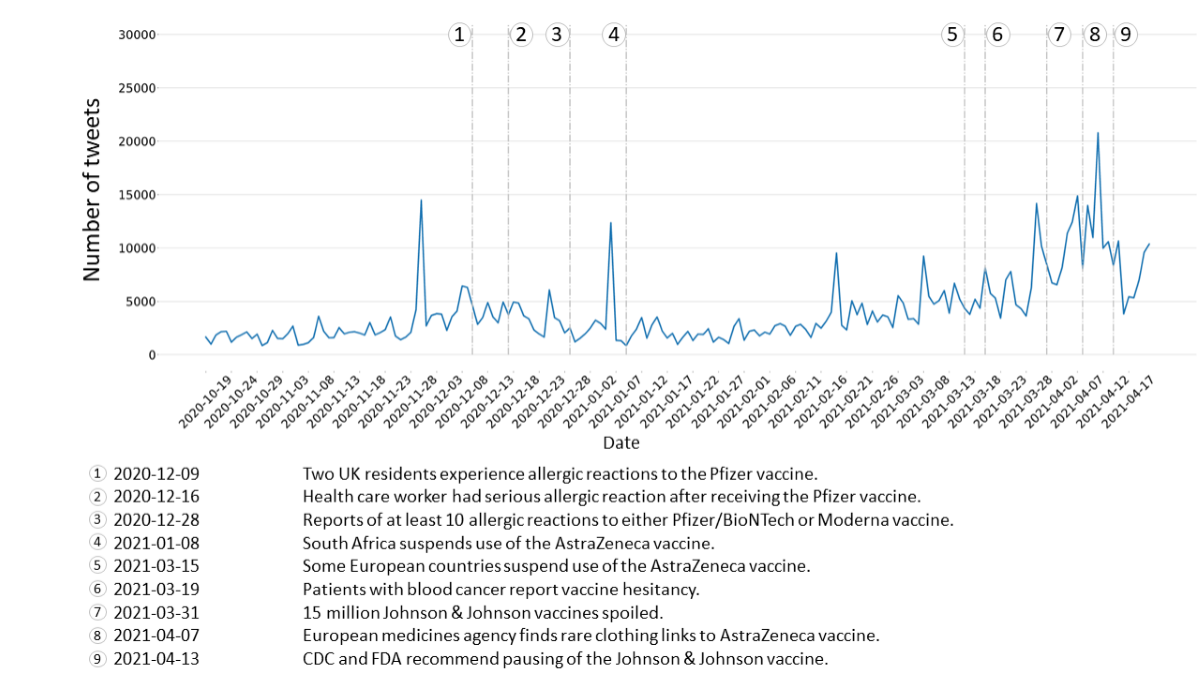
Number of tweets over time in the streaming collection. The times of adverse events related to vaccines are marked by dashed vertical lines. Further descriptions of the news items are provided in the legend below the chart. CDC: US Centers for Disease Control and Prevention; FDA: US Food and Drug Administration.

The streaming collection consists of 1.8 million tweets created by 719,000 unique accounts between October 18, 2020, and April 21, 2021. As shown in Figure 1, the number of relevant tweets in the streaming collection gradually increases from the start date. The chatter is relatively stable, with small spikes that do not often correspond to major announcements regarding vaccine research or vaccine authorization. We find this surprising, as the news usually drives the discussion on Twitter. Additionally, we observed a large spike in activity near the end of November 2020 that was not caused by any single event but rather by the increased activity of a small number of accounts.

**Figure 2:**
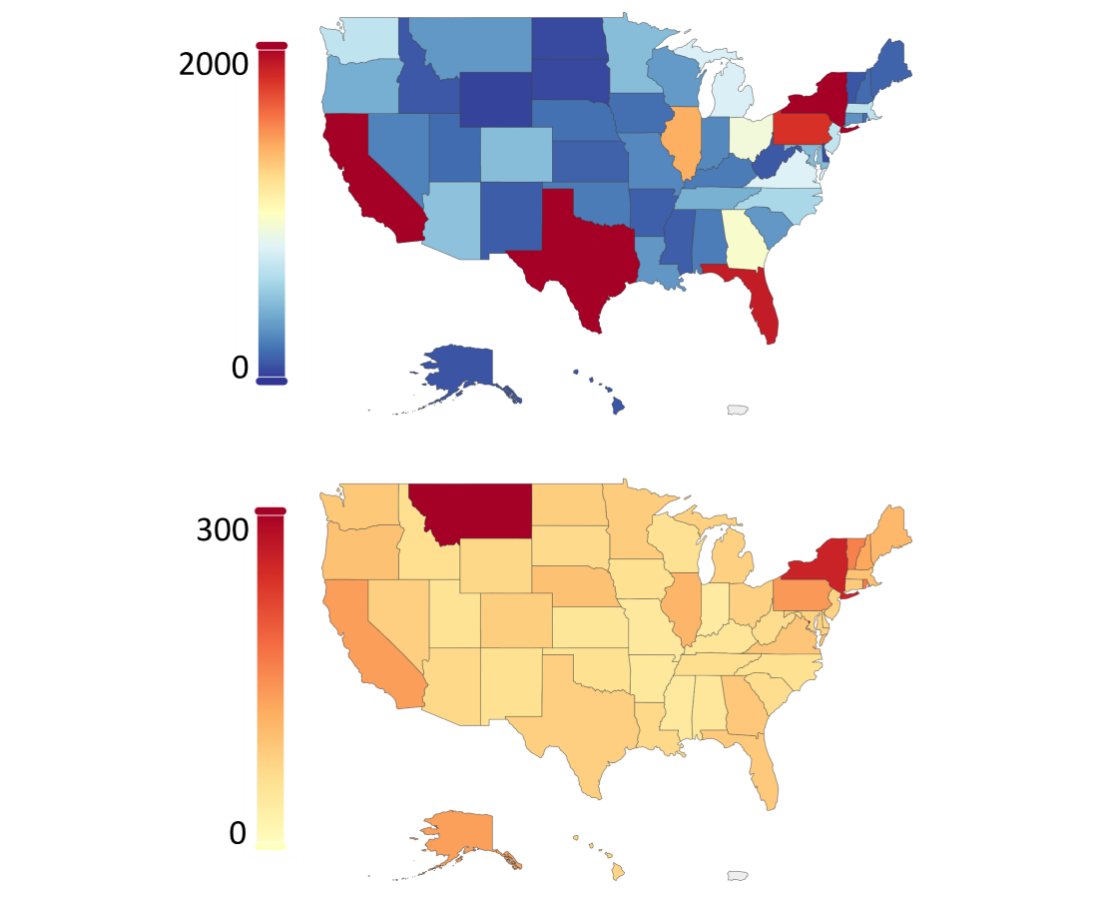
Geographical distribution of the tweets from the streaming collection originating in the United States. The location of the tweets was inferred from the self-reported location of the account. Top: absolute number of tweets in each state; bottom: number of tweets normalized by the state population.

The overwhelming majority of tweets originated from countries with predominantly English-speaking populations. Out of 1,832,333 tweets in the streaming collection, 1,245,986 (68%) originated in the United States, 229,041 (12.5%) in Great Britain, 100,778 (5.5%) in Canada, 21,987 (1.2%) in Ireland, and 20,155 (1.1%) in Australia; the rest of the tweets originated from other countries. In Figure 2, we show the geographical distribution of tweets in the United States. As expected, states with a large population, such as California, Texas, Florida, and New York, have more tweets in absolute terms (Figure 2, top). The number of tweets normalized by state population is depicted in Figure 2 (bottom), with the most tweets per capita originating from Hawaii, Alaska, and Maine, respectively.

Table 3 lists the top 15 most tweeted hashtags in the streaming collection. The count column represents the total number of times a hashtag appears, and the proportion column quantifies the proportion of tweets that contain a specific hashtag out of all tweets with any hashtag. Note that many tweets contain no hashtags, and many tweets with a hashtag contain more than one hashtag. In addition to the most common general hashtags that we expected to find, such as *#vaccine* and *#covid19*, we observed a high proportion of hashtags that carry strong antivaccine sentiment, such as *#novaccineforme*, *#vaxxed* and *#vaccineinjury*. For example, *#novaccineforme* can be found in more than 25,000 tweets, accounting for 6.6% of all tweets in the streaming collection that contain any hashtags. A large set of common hashtags is related to some debunked conspiracy theories that claim there is a global plot by rich individuals to reduce the world population, often expressed through hashtags such as *#depopulation*, *#billgatesbioterrorist* and *#arrestbillgates*. Another set of very frequent hashtags appears benign on the surface. Hashtags such as *#learntherisk* and *#informedconsent* appear to communicate genuine concerns about the safety of the vaccines; however, those hashtags are usually decoys and are very often used by the same accounts that strongly oppose vaccination and that otherwise often use more explicit antivaccine hashtags.

**Table 3:** Top 15 hashtags in the streaming data set. The count is the total number of times a hashtag appears, and the proportion quantifies the proportion of tweets that contain a specific hashtag out of all tweets with a hashtag.

Hashtag	Count, n	Proportion (%)
vaccine	41,069	10.66
vaccines	33,050	8.58
covid19	26,616	6.91
novaccineforme	25,642	6.66
learntherisk	23,340	6.06
billgatesbioterrorist	20,197	5.24
study	20,166	5.23
novaccine	19,410	5.04
mybodymychoice	19,166	4.97
informedconsent	16,578	4.30
depopulation	15,021	3.90
vaxxed	12,691	3.29
vaccineinjury	12,640	3.28
vaccination	10,873	2.82
arrestbillgates	9991	2.59

### Account Collection

**Figure 3:**
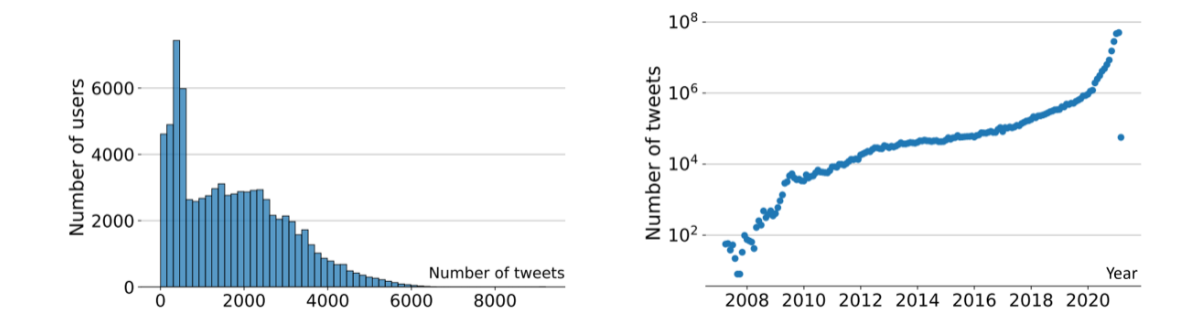
Tweets in the account collection. Left: distribution of tweets per account; right: distribution of tweets over time.

The account collection differs from the streaming collection, as it is focused on historical tweets from a set of accounts. The process of collecting the historical tweets is explained more in detail in the *Methods* section. The current account collection consists of more than 135 million tweets published by over 78,000 unique accounts, and it spans the period from March 3, 2007, to February 8, 2021. In Figure 3, we illustrate some of the most important statistics from this data collection. The left panel in Figure 3 shows the distribution of the number of tweets per account. Out of 78,954 accounts, 39,350 (49.8%) published fewer than 1500 tweets, 31,581 (40%) of the accounts have more than 2000 tweets, and 1184 (1.5%) have more than 5000 tweets. The right panel in Figure 3 shows the number of tweets over time. Most of the tweets originate in the year 2020, with the oldest tweet dating back to 2007. For 55,267 (70%) of the 78,954 accounts, the oldest collected tweet dates from 2020. There is a significant portion of accounts whose historical tweets date much earlier; for 14,211 (18%) of the 78,954 accounts, the earliest tweet was dated before 2018, and for 5368 (6.8%) of the accounts, the earliest tweet was dated before 2014. This relatively long-spanning collection of historical tweets at the account level may allow for a comprehensive temporal analysis of vaccine hesitancy development on Twitter over several years.

The 15 most common hashtags appearing in the account collection are displayed in Table 4. In addition to the common COVID-19–related hashtags, we observe many hashtags referring to US politics. During the period of the US 2020 presidential election and the political campaign, the accounts that appear in our collection were particularly active. Hence, we can see that many politically motivated narratives in the data originated during that period.

**Table 4:** Top 15 hashtags in the account collection. The count is the total number of times a hashtag appears, and the proportion quantifies the proportion of tweets that contain a specific hashtag out of all tweets with a hashtag.

Hashtag	Count	Proportion (%)
covid19	474,481	2.55
endsars	203,297	1.09
maga	164,332	0.88
coronavirus	158,574	0.85
trump	156,262	0.84
stopthesteal	121,069	0.65
trump2020	115,002	0.62
breaking	111,274	0.60
obamagate	110,046	0.59
covid	106,095	0.57
china	98,026	0.53
oann	96,943	0.52
antifa	79,157	0.43
biden	77,728	0.42
fakenews	66,599	0.36

### News Sources in the Streaming Collection

**Figure 4:**
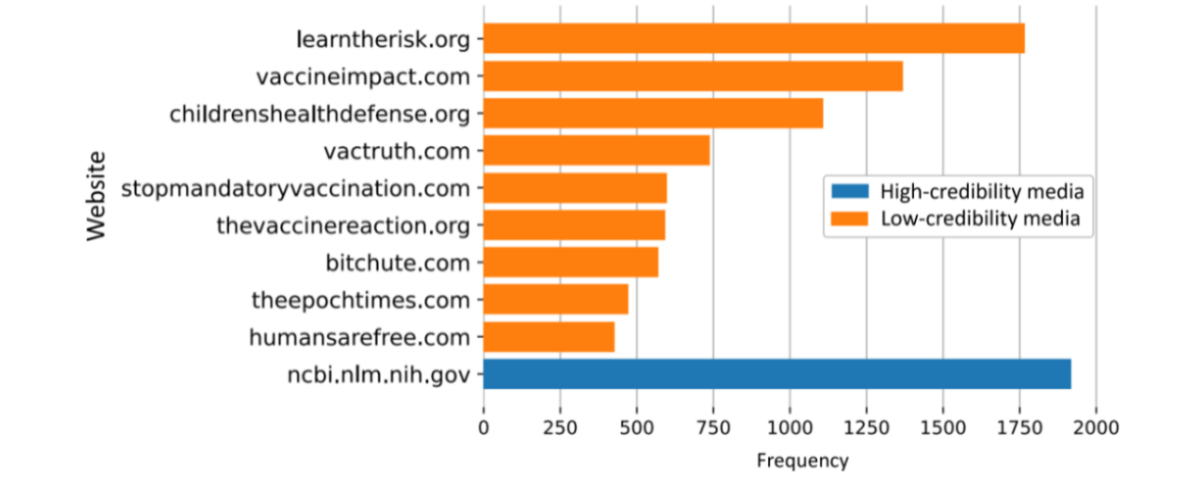
Top 10 news sources in the streaming collection. The URLs of the news aggregators and the large social platforms were omitted.

Vaccine hesitancy is usually fueled by misinformation originating from websites with questionable credibility. In Figure 4, we list the top 10 URLs that can be found in the streaming collection, and we illustrate the number of times each appears. The vast majority of those websites can be found in the Iffy+ database of low credibility sites [20]. One of the most commonly shared sources is the website of an American antivaccine group called Learn The Risk; it is known for its campaigns against vaccination, which assert that vaccines are responsible for a large number of deaths of young children. It is followed by Vaccine Impact, a well-known news and information website that promotes pseudoscience; this website often shares antivaccination propaganda and promotes alternative medicine, holism, and alternative nutrition. The only website on the list with high credibility is the website of the National Center for Biotechnology Information (NCBI), a PubMed parent company.

### News Sources in the Account Collection

**Figure 5:**
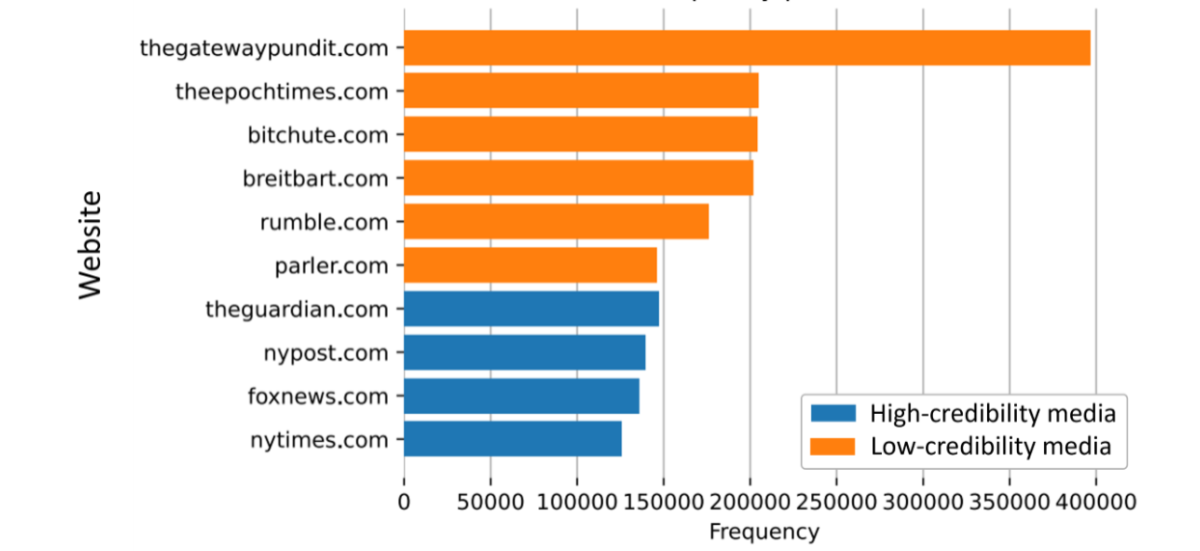
Top 10 URLs in the account collection. The URLs of the news aggregators and the large social platforms were omitted.

In Figure 5, we list the top 10 URLs that can be found in the account collection, and we illustrate the number of times each appears. Figure 5 shows that many far-right news media sites appear frequently in the account collection. The Gateway Pundit [25], which is known for publishing falsehoods, hoaxes, and conspiracy theories, occurs more than 400,000 times. Other far-right media outlets, such as Breitbart News [26] and the Epoch Times [27], also appear very often. Considering the sources that usually fall in the group of mainstream news media sites, such as Fox News [28] and the *New York Post* [29], conspiracy spreaders selectively quote reports from these sources to increase the credibility of often false claims.

### Political Leanings of the Antivaccination Accounts

**Figure 6:**
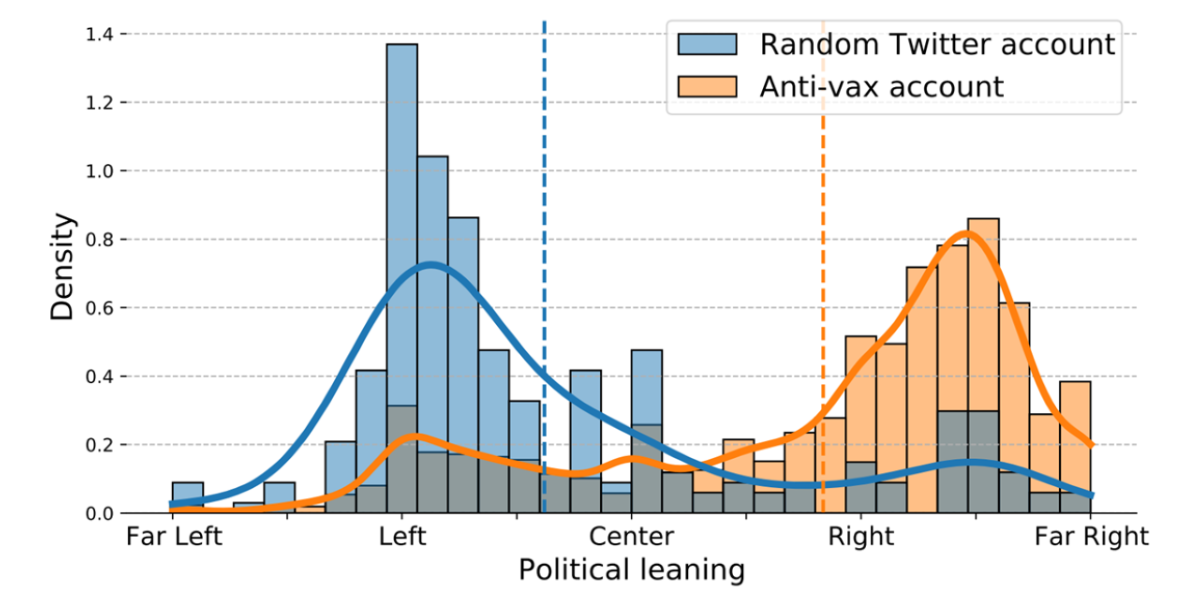
Distributions of the Twitter accounts based on their political leaning and attitude toward vaccination. The political leaning of each account was calculated from its media diet. Anti-vax: antivaccination.

In Figure 6, we show the distribution of political leanings of the accounts. The political leaning of an account was estimated based on its media diet (see the *Methods* section). The x-axis represents the account’s political leaning and can take any value between “far left” and “far right.” The y-axis is the normalized number of accounts with a corresponding political leaning. The political leaning of the accounts engaged in the antivaccination narratives is shown in orange. We observed a bimodal distribution with a significantly higher right peak. The blue bars illustrate the distribution of the political leanings for random Twitter accounts. The random Twitter accounts are a random sample of approximately 6000 accounts from the previously published Twitter data set related to the US 2020 Presidential election by Chen et al [30]. It has been previously shown that the Twitter users are younger on average and more likely to vote Democrat than the general public [31,32]. These results are not surprising, as they align with earlier studies showing that political orientation is a strong predictor of vaccine hesitancy in the United States [33,34].

### Clusters of Antivaccine Narratives in the Streaming Collection

**Figure 7:**
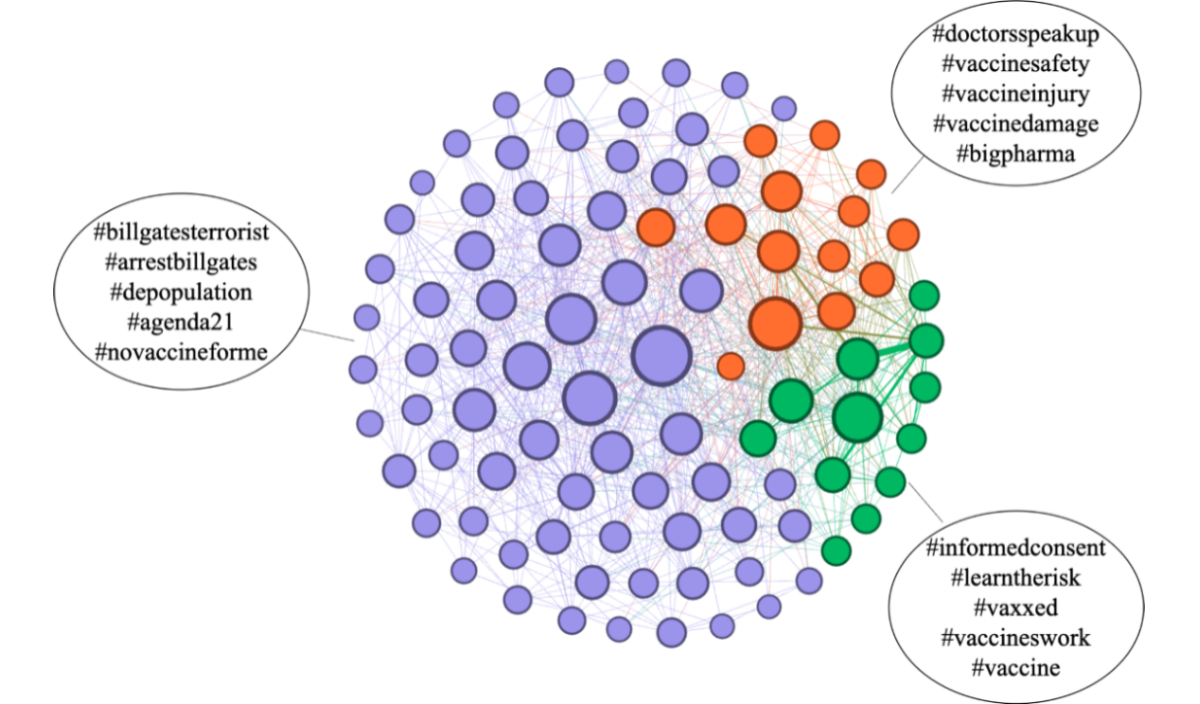
An overview of the prominent hashtags in the data set, clustered into 3 communities. The nodes are the hashtags, and the links are drawn between two hashtags that appear together in the same tweet. Clustering was performed using the Louvain algorithm. For readability, we do not show all the node labels.

To obtain further insights into the provided data set, we explored the clusters of antivaccine narratives by identifying the antivaccine topics that usually co-occurred. We ran the Louvain community detection algorithm on the topic co-occurrence network, as described in the *Methods* section. The topic network is illustrated in Figure 7. We identified 3 distinct communities; all of them contained antivaccine keywords, but with different focuses on topics. The largest topic community, colored purple, focuses on debunked claims around the conspiracy narrative that the vaccine is a plot by rich people to reduce the world population. The second topic community, colored orange, mostly focuses on vaccine safety, as hashtags such as *#doctorsspeakup*, *#vaccinesafety*, and *#vaccineinjury* appear often. The smallest topic community, in green, contains a mixture of various hashtags that range from strongly antivaccine, such as *#informedconsent*, *#learntherisk*, and *#vaxxed*, to some neutral hashtags, such as *#vaccine*, to some provaccine hashtags, such as *#vaccineswork*.

## Discussion

### Principal Findings

In this paper, we present a comprehensive data set consisting of tweets related to antivaccination narratives, organized in streaming and account collections. We characterized the data in several ways, including frequencies of prominent keywords, news sources, geographical location of the accounts, and political leaning of the accounts. The streaming collection consists of a random sample of tweets that contain any of the specific keywords promoting strong antivaccination sentiments. This is a common method used to collect Twitter data on vaccination hesitancy and other similar topics [35-42]. It is well understood by academics and is often used to provide useful insights about the chatter on the web about a particular topic in a specific period. The account collection was gathered using a relatively new method of collecting Twitter data by querying the historical activities from a set of tracked accounts. This collection was made possible after Twitter introduced the Academic Research product track API. In this way, by gathering massive amounts of historical tweets, researchers can characterize individual accounts rather than populations on average. This data set will be useful for scientists interested in the demographic and psychographic characteristics of Twitter users who are prone to spreading antivaccination narratives.

The news sources shared by the users in the streaming collection are predominantly websites with low credibility. However, the most shared URL is the website of the NCBI [25], which is part of the United States National Library of Medicine, a branch of the National Institutes of Health. NCBI houses PubMed, the largest bibliographic database for biomedical literature. This finding can create a false impression that the tweets from the streaming collection contain information from legitimate scientific sources. When we examined the context in which those papers were shared, we discovered that most of the papers from PubMed were cited with false and misleading conclusions. Sometimes, antivaccine advocates would share legitimate scientific papers documenting rare side effects of the vaccines, while overemphasizing the observed adverse effects and calling for vaccine boycotts. Sharing a scientific study in a tweet provides an illusion of credibility. Cherry-picking desirable sentences and relying on the fact that most of the audience will not make an effort to read a scientific paper in detail is a very effective strategy for manipulation.

It is often valuable to know the political affiliation of users who share antivaccine narratives. Knowing users’ position on a political spectrum can be useful in identifying their most likely moral values and possible stances toward specific societal issues. This knowledge can be used to design appropriate future messaging and campaigns. We were able to identify the political affiliation for the accounts collection, as we had enough tweets for each account. Accounts that share common misinformation related to vaccines often share other conspiracy narratives, usually politically charged ones. The population susceptible to such narratives strongly skews conservative [18]; therefore, we expected that a large number of accounts in the account collection would be right leaning.

### Limitations

Although the data sets give an overview of vaccine hesitancy on Twitter, potential limitations warrant some considerations. First, our streaming collection relies on a defined set of keywords. The antivaccine lingo is constantly evolving as the COVID-19 pandemic unfolds. Although we have made our best efforts to find the most representative keywords, they may not fully cover all antivaccine topics. The set of keywords we used was designed to capture the strongest antivaccine sentiments and may have missed various nuances in the multifaceted nature of vaccine hesitancy. Second, this data set should not be used to draw conclusions for the general population, as the Twitter user population is younger and more politically engaged than the general public [31]; this means that our data may be biased in various ways. Additionally, the keywords used for the collection were derived from the English vocabulary, highly biasing the geographical distribution of the tweets toward the English-speaking regions of the world. Finally, to prevent the spread of misleading COVID-19 information, Twitter has enacted specific rules and policies. The accounts violating these rules and policies may be banned by Twitter, making their tweets unreachable. At the time of writing, our estimate is that more than 40% of the accounts in the streaming collection and 30% of accounts in the accounts collection had been either banned or deleted. With each update of the streaming data set, we expect this proportion to change.

### Conclusion

In addition to the streaming collection, which tracks tweets as they appear in real time, perhaps the most important contribution of this study is the account collection, a data set consisting of almost all historical tweets for a sample of users who were actively sharing antivaccination narratives. This data set can be used to provide further insights into the accounts that engage in antivaccine propaganda. Our intention in publishing this paper and data sets is to provide researchers with assets to enable further exploration of issues revolving around vaccine hesitancy and to study them through the lens of social media. The data sets collected and provided here could be useful for researchers interested in tracking the longitudinal characteristics of accounts engaging with antivaccine narratives. It can help provide better insights into the socioeconomic, political, and cultural determinants of vaccine hesitancy.

### Use Notes

The data set is released in compliance with the Twitter Terms and Conditions and the Developer’s Agreement and Policies [12]. Researchers who wish to use this data set must agree to abide by the stipulations stated in the associated license and conform to Twitter’s policies and regulations.

### Data Availability

The data are available at GitHub [15].

## Abbreviations

API: application programming interface
DARPA: Defense Advanced Research Projects Agency
NCBI: National Center for Biotechnology Information
